# Repulsive Guidance Molecule a Inhibits Angiogenesis by Downregulating VEGF and Phosphorylated Focal Adhesion Kinase *In Vitro*

**DOI:** 10.3389/fneur.2017.00504

**Published:** 2017-09-26

**Authors:** Gang Zhang, Rong Wang, Ke Cheng, Qi Li, Yu Wang, Rongrong Zhang, Xinyue Qin

**Affiliations:** ^1^Department of Neurology, The First Affiliated Hospital of Chongqing Medical University, Chongqing, China; ^2^Department of Gastrointestinal Surgery, The First Affiliated Hospital of Chongqing Medical University, Chongqing, China; ^3^Department of Neurosurgery, The First Affiliated Hospital of Chongqing Medical University, Chongqing, China

**Keywords:** repulsive guidance molecule a, VEGF, phosphorylated focal adhesion kinase, angiogenesis, neogenin

## Abstract

Repulsive guidance molecule a (RGMa) is a major neuron guidance factor in central nervous systems. We previously found that inhibition of RGMa could greatly enhance neural function rehabilitation in rats after MCAO/reperfusion. Neuron guidance factors are often regulators of angiogenesis. However, the effect of RGMa on angiogenesis and its mechanisms remain to be determined. Here, we investigated the effect of RGMa on endothelial cell (EC) proliferation, migration, tube formation, and cytoskeleton reassembly. The addition of recombinant RGMa significantly decreased the proliferation, migration, and tube formation of ECs. It also decreased the level of phosphorylated focal adhesion kinase (p-FAK Tyr397). Furthermore, the F-actin of the cytoskeleton assembly was obviously suppressed, with decreased filopodia and lamellipodia after the addition of RGMa. Knockout of neogenin or Unc5b significantly diminished RGMa’s inhibition of EC migration, tube formation, and cytoskeleton reassembly. RGMa-induced p-FAK (Tyr397) decrease was also abolished by knockout of neogenin or Unc5b. These results indicate that RGMa may be a negative regulator of angiogenesis through downregulating VEGF and p-FAK (Tyr397) *via* neogenin and Unc5b *in vitro*.

## Introduction

Acute ischemic stroke is a leading cause of severe cognitive impairment, physical disability, and mortality worldwide. Therapies for ischemic stroke have made enormous progress in revascularization, such as intravenous thrombolysis and intravascular intervention therapy; however, most patients are not suitable for these effective treatments due to the narrow therapeutic time window. For this reason, novel effective approaches for stroke therapy need to be explored. Angiogenesis is an important strategy for poststroke rehabilitation because sufficient perfusion can provide the requisite molecules for recovering neural networks (1) and can contribute to the clearing of cell debris (2, 3).

Repulsive guidance molecules (RGMs) were originally identified as membrane-bound proteins that function as axon-repellent guidance molecules in the chick nervous system (4). Repulsive guidance molecule a (RGMa) is a key regulator of many cell processes, including neural guidance (5), cell differentiation, migration, and adhesion (6). RGMa may also be involved in the pathogenesis of many diseases, such as Parkinson’s, Alzheimer’s, multiple sclerosis, and spinal cord injury (7–9). We previously found that RGMa was significantly increased and induced growth-cone collapse in an MCAO/reperfusion rat model (10). Moreover, we found that minocycline, RNA interference, and olfactory bulb stimulation significantly reduced the expression of RGMa and promoted neuronal functional recovery after acute cerebral ischemia (11–13). Our previous study demonstrated that RGMa induced growth-cone collapse *via* activation of the ROCK/CRMP-2 and GSK-3β/CRMP-2 pathways (10). Bryan et al. reported that RhoA/ROCK signaling is involved in many aspects of VEGF-induced angiogenesis (14). Because the nervous and vascular systems are organized in remarkably parallel ways (15), axon guidance and blood vessel patterns share the same cues and receptors (16–18). Thus, we speculate that RGMa may be a regulator of angiogenesis. Yamashita et al. reported that RGMa suppressed angiogenesis of human umbilical artery endothelial cells (HUAECs) *in vitro* and in a Matrigel plug model *in vivo* (19). However, whether RGMa suppresses angiogenesis in other endothelial cells (ECs), especially brain microvascular ECs, and the mechanisms of RGMa in angiogenesis remain unclear. The present study aimed to explore the role and mechanisms of RGMa in angiogenesis.

Neural guidance factors, such as netrins, semaphorins, ephrins, and slits, play critical roles in regulating angiogenesis (16–18). Netrin-1 and netrin-4 were involved in antiangiogenesis *via* binding to neogenin (20, 21). Unc5b was an essential co-receptor during the inhibition of angiogenesis and phosphorylation of focal adhesion kinase (FAK) induced by netrin-1 and netrin-4 in ECs (21). Unc5b was also confirmed as a repulsive factor in ECs during angiogenesis (22). Neogenin and Unc5b are essential for RGMa-induced axon-core collapse (23). Therefore, we speculate that RGMa may affect angiogenesis by involvement of neogenin and Unc5b.

## Results

### RGMa Expression Increased in HUAECs, Human Umbilical Vein Endothelial Cells (HUVECs), and Rat Brain Microvascular Endothelial Cells (RBMECs) Stimulated with VEGF

**Figure 1:**
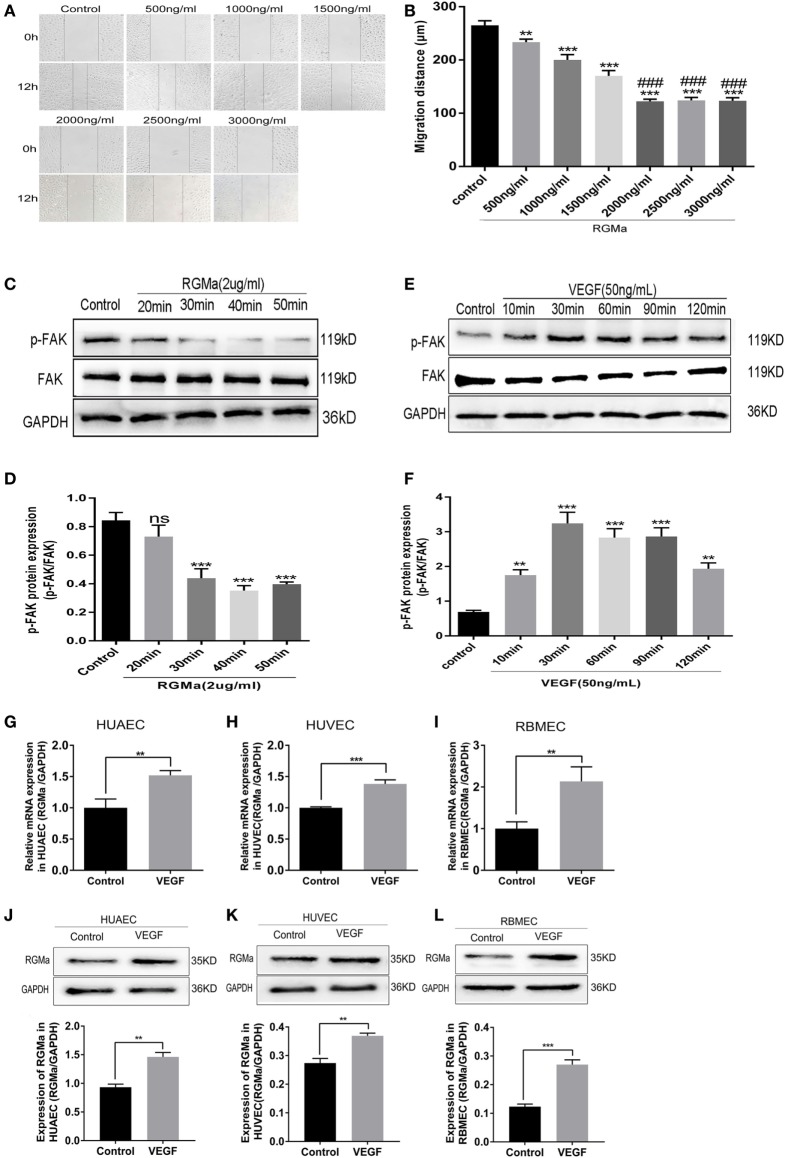
Repulsive guidance molecule a (RGMa) expression increased in human umbilical artery endothelial cells (HUAECs), human umbilical vein endothelial cells (HUVECs), and rat brain microvascular endothelial cells (RBMECs) stimulated with VEGF. **(A,B)** Endothelial cell (EC) migration distance was evaluated by scratch assay. ECs were treated with a dose curve of RGMa (500–3000 ng/ml), and images were taken at the beginning and 12 h. **(C,D)** ECs were treated with RGMa (2 µg/ml), and lysates were collected over a time course of 50 min. The amount of phosphorylated focal adhesion kinase (p-FAK) protein was visualized with western blot analysis. **(E,F)** ECs were treated with VEGF (50 ng/ml), and lysates were collected over a course of 120 min. p-FAK protein levels were visualized with western blot analysis. **(G–I)** Quantitative real-time polymerase chain reaction showed RGMa mRNA level was upregulated in HUAECs, HUVECs, and RBMECs exposed to VEGF (50 ng/ml) for 30 min. **(J–L)** RGMa protein levels were visualized in HUAECs, HUVECs, and RBMECs exposed to VEGF at 30 min by western blot analysis. Data in bar graphs represent the means ± SD of ≥4 independent experiments. *P < 0.05, **P < 0.01, ***P < 0.001, ^#^VS 1500 ng/ml, ^###^*P* < 0.001.

The dose curve of RGMa on HUAEC migration was measured with a scratch assay. As shown in Figure 1A, the HUAEC migration distance gradually decreased with increased RGMa concentration. When the concentration of RGMa was ≥2 µg/ml, the cell migration distance did not continue to decrease with the increased concentration. Thus, we used an RGMa concentration of 2 µg/ml for the follow-up experiments (Figure 1B). To test whether RGMa treatment results in decrease of p-FAK (Tyr397) in ECs, the level of p-FAK (Tyr397) was examined over time with RGMa stimulation by western blot. Within 40 min of RGMa stimulation, p-FAK (Tyr397) protein levels decreased by half, then began to increase after 50 min (Figures 1C,D). Over a time course of VEGF stimulation, p-FAK (Tyr397) level increased by ≥3-fold, then began to decrease after 90 min, but was still almost 2-fold greater than in the control (Figures 1E,F). RGMa mRNA (Figures 1G–I) and protein levels (Figures 1J–L) were significantly increased in ECs after the addition of VEGF for 40 min. These findings indicate that RGMa may be a negative regulator in angiogenesis.

### RGMa Decrease VEGF Expression, p-FAK (Tyr397), Proliferation, Migration, and Tube Formation in ECs

**Figure 2:**
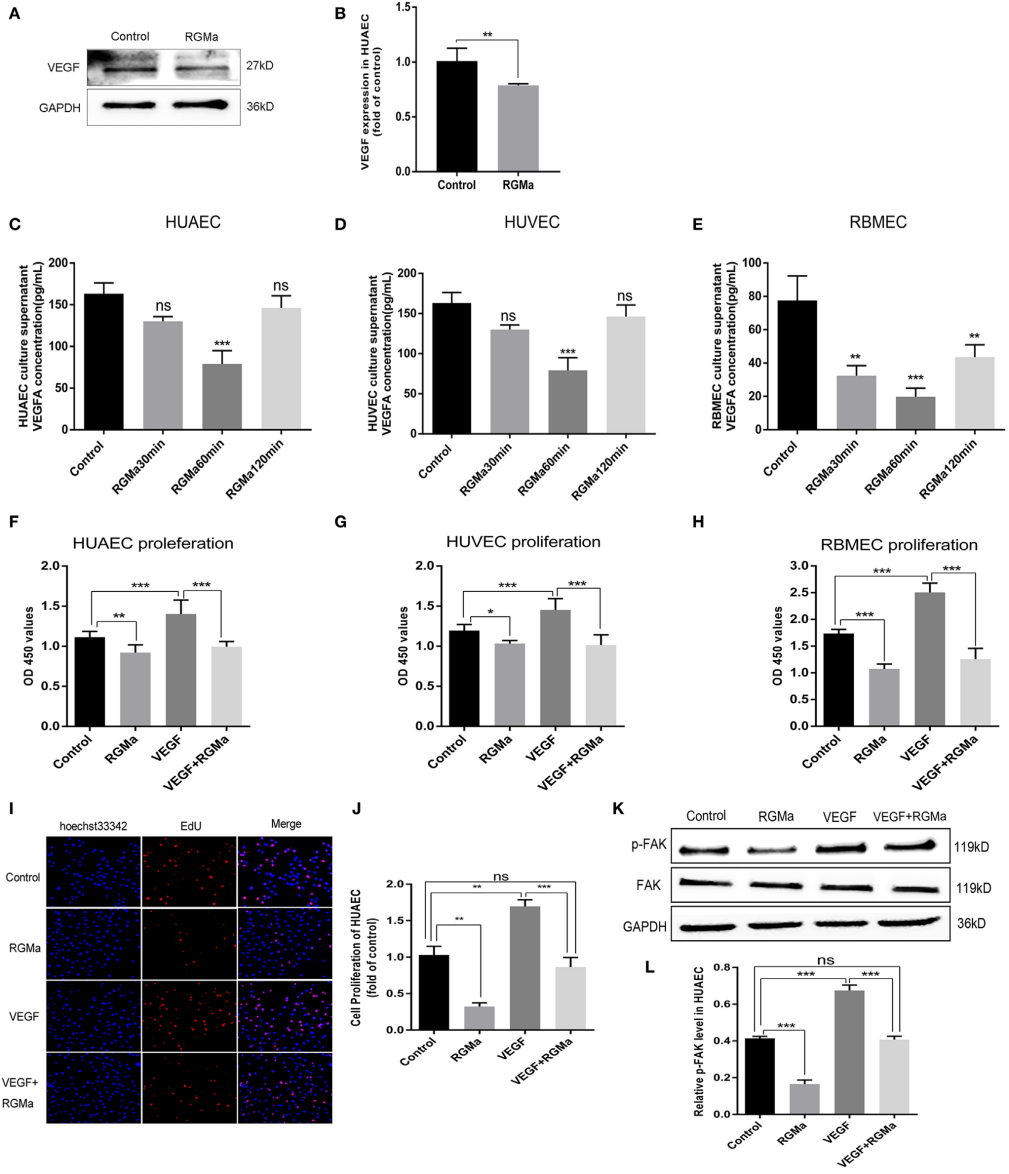

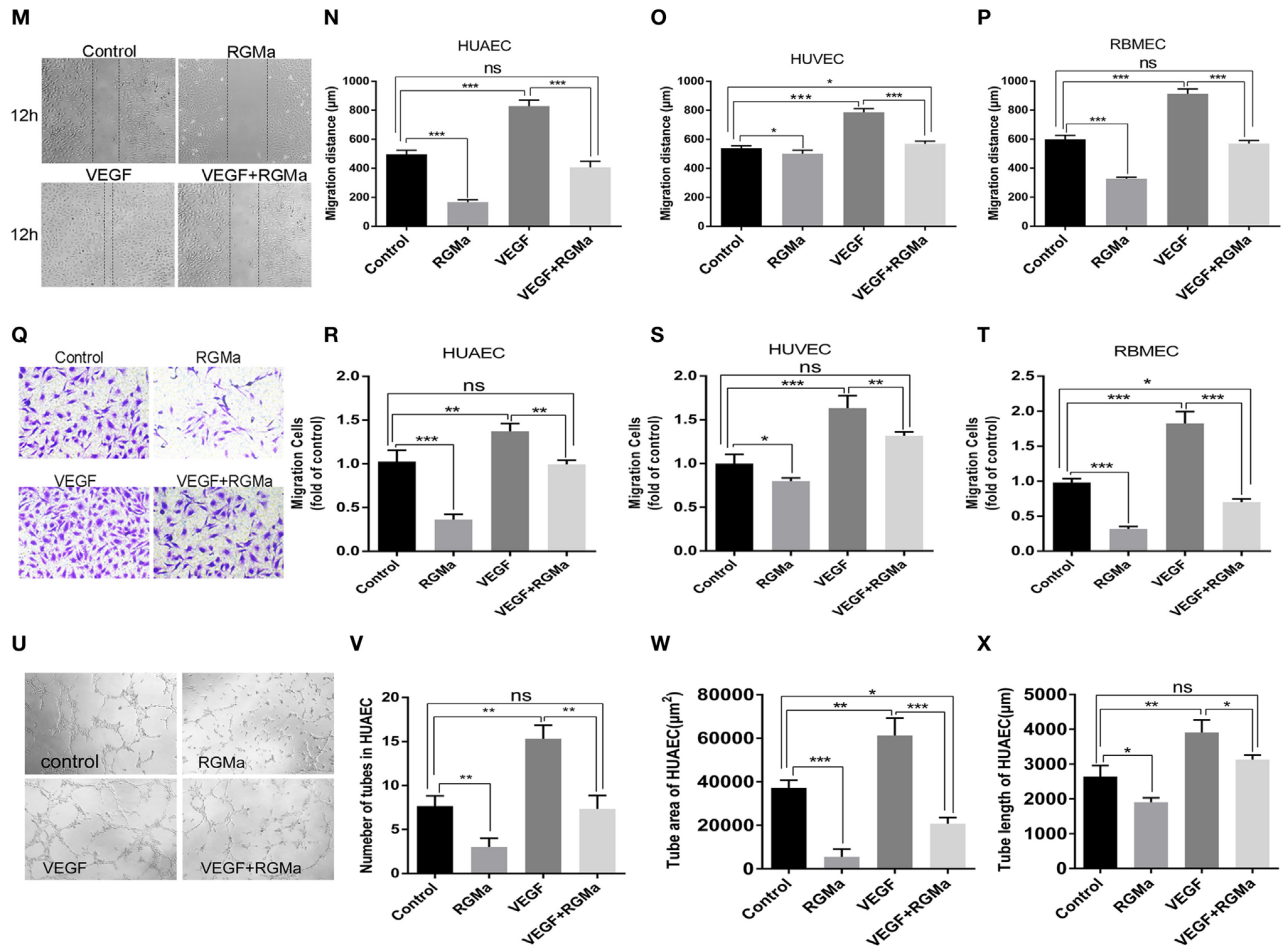
Repulsive guidance molecule a (RGMa) suppressed VEGF expression, phosphorylation of focal adhesion kinase (FAK), proliferation, migration, and tube formation in ECs. **(A,B)** Lysate was collected, and VEGF was detected by western blot in human umbilical artery endothelial cells (HUAECs) treated with RGMa (2 µg/ml); **(C–E)** ELISA kit assay showed VEGFA decreased in endothelial cell (EC)-culture supernatant exposed to RGMa compared with cell-culture supernatant from control group. **(F–H)** Cell proliferation was evaluated with cell-counting kit-8 and 5-ethynyl-2′-deoxyuridine (EdU) assays **(I,J)**. RGMa decreased proliferation of ECs stimulated and unstimulated with VEGF. **(K,L)** FAK (Tyr397) phosphorylation was measured with western blot in HUAECs treated with vehicle, RGMa (2 µg/ml), VEGF (50 ng/ml), or VEGF plus RGMa. **(M–P)** ECs were grown to 100% confluence, serum-starved overnight, wounded with a sterile pipette tip to remove cells, and treated with control, RGMa, VEGF, or VEGF plus RGMa. Photographs (40×) were taken at 12 h after injury. Wound closure of ≥3 wells was quantified and reported as mean ± SD. **(Q–T)** Migration activity of ECs treated with RGMa, VEGF, VEGF plus RGMa, or control was measured with transwell assay. Photographs (200×) were taken 18 h after treatment. **(U–X)** HUAECs were starved overnight, treated as indicated, and seeded into 96-well plates coated with Matrigel. Photographs (40×) were taken at 3 h after treatment. The number of tubes, tube area, and tube length were analyzed with Image J. Scale bar, 100 µm. Data shown are representative of experimental and quantitative results. *N* ≥ 4 independent experiments. Bars represent mean ± SD. *P < 0.05, **P < 0.01, ***P < 0.001.

To explore the possible mechanism of RGMa in angiogenesis, intracellular VEGF protein levels in HUAECs and VEGFA concentrations in cell-culture supernatants from ECs were separately detected with western blot and ELISA kits. VEGF protein expression was significantly decreased in HUAECs treated with RGMa when compared with the control group (Figures 2A,B). The cell-culture supernatants were collected from the control and RGMa groups at 30, 60, and 120 min. ELISA assays showed that VEGFA was greatly decreased in the RGMa group compare with the control group (Figures 2C,E). p-FAK (Tyr397) levels in HUAECs were evaluated in the control, RGMa (2 µg/ml), VEGF (50 ng/ml), and RGMa-plus-VEGF groups. FAK phosphorylation (Tyr397) levels in HUAECs treated with RGMa were significantly decreased compared with those of the control group. FAK phosphorylation (Tyr397) levels in HUAECs treated with RGMa plus VEGF was significantly decreased compared to the VEGF group (*P* < 0.01) (Figures 2K,L). Meanwhile, proliferation (*P* < 0.05) (Figures 2F–J), migration (*P* < 0.05) (Figures 2M–T), and tube formation (*P* < 0.05) (Figures 2U–X) in HUAECs treated with or without VEGF were also significantly attenuated by the addition of RGMa. These results suggest inhibit effects of RGMa *in vitro* angiogenesis may through downregulate VEGF and FAK phosphorylation (Tyr397).

### RGMa Inhibited Angiogenesis *In Vitro via* Neogenin

**Figure 3:**
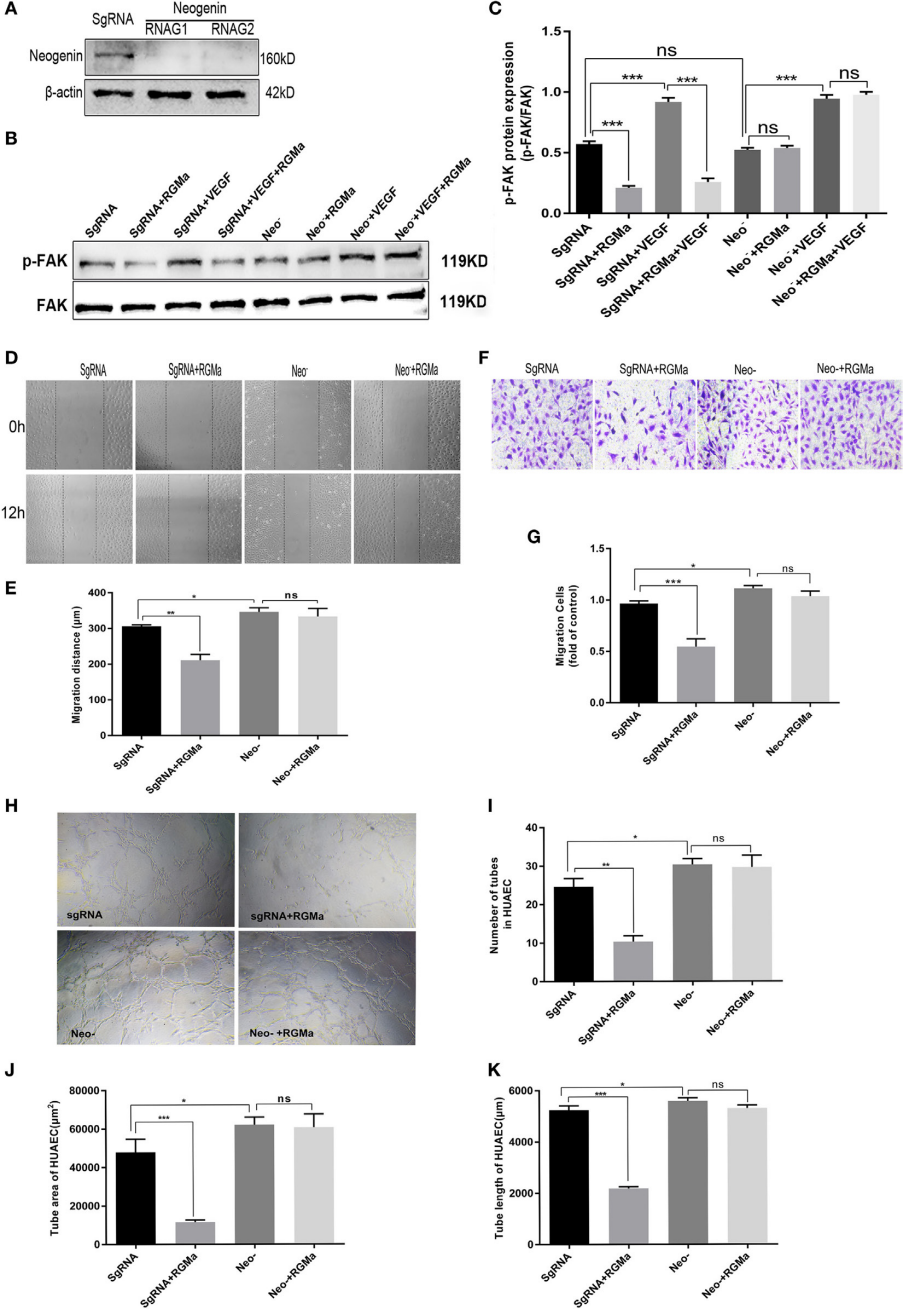
Repulsive guidance molecule a (RGMa) inhibited angiogenesis *in vitro via* neogenin. **(A)** Human umbilical artery endothelial cells (HUAECs) were transfected with CRISPR/Cas9 neogenin knockout kit and purified with puromycin, then the result of neogenin knockout was validated with western blot. **(B,C)** HUAECs transfected with SgRNA or neogenin gRNA were treated with vehicle, RGMa, VEGF, or VEGF plus RGMa. The focal adhesion kinase (FAK) (Tyr397) phosphorylation was measured with western blot. **(D–G)** Migration and **(H–K)** tube formation of HUAECs transfected with SgRNA or neogenin gRNA were determined by scratch, transwell, and Matrigel tube-formation assays. The relative number of tubes, tube area, and tube length were analyzed with Image J. Scale bar, 100 µm. Data shown are representative of experimental and quantitative results. *N* ≥ 4 independent experiments. Bars represent mean ± SD. *P < 0.05, **P < 0.01, ***P < 0.001.

To verify that RGMa inhibits p-FAK signaling through the receptor neogenin, we established knockout cells for neogenin with two kinds of plasmids containing different RNAG sequences (RNAG1 and RNAG2). Whereas scrambled RNAG (negative control) was ineffective in reducing neogenin expression (Figure 3A). Using this knockout technique, we observed that neogenin knockout significantly blocked RGMa-induced decreases in *p-FAK (Tyr397)* levels (*P* < 0.01), as shown in Figures 3B,C. EC migration distance (Figures 3D,E) (*P* < 0.05), EC migration number (Figures 3F,G), and tube formation (*P* < 0.05) (Figures 3H–K) were significantly decreased by RGMa in HUAECs transfected with SgRNA, but this inhibition effect was completely blocked by neogenin knockout. Neogenin knockout increased EC migration distance, EC migration number, and tube-formation ability compared with ECs transfected with SgRNA. These results indicate that RGMa inhibited *in vitro* angiogenesis by downregulated p-FAK (Tyr397) *via* neogenin in ECs.

### Unc5b Was Involved in the Effects of RGMa on Phosphorylation of FAK, Migration, and Tube Formation in HUAECs

**Figure 4:**
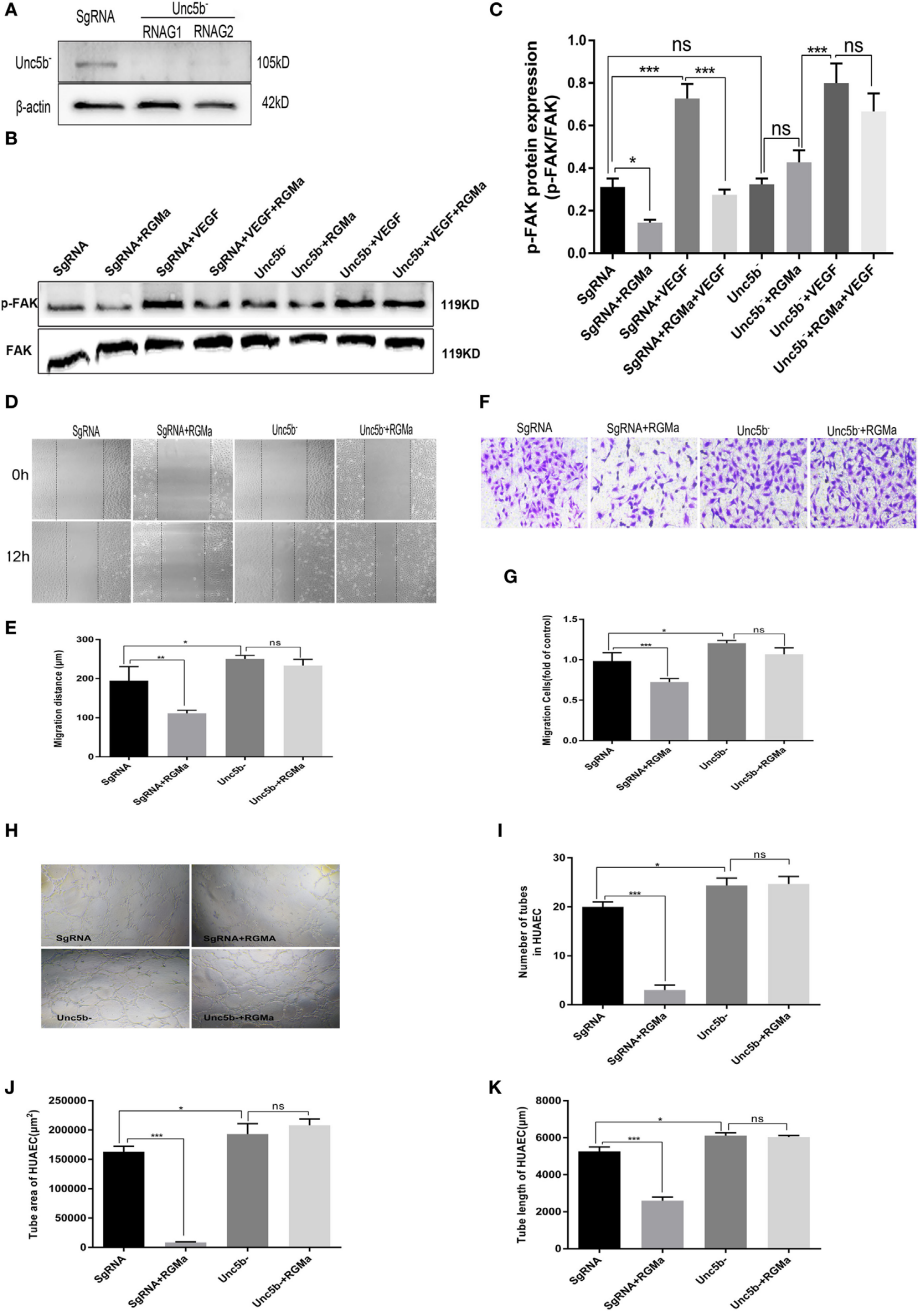
Unc5b is involved in the effect of repulsive guidance molecule a (RGMa) on phosphorylation of focal adhesion kinase (FAK), migration, and tube formation in human umbilical artery endothelial cells (HUAECs). **(A)** HUAECs were transfected with CRISPR/Cas9 Unc5b knockout kits and purified with puromycin, then the effect of Unc5b knockout was validated with western blot. **(B,C)** HUAECs transfected with SgRNA or Unc5b gRNA were treated with control, RGMa, VEGF, or VEGF plus RGMa. FAK (Tyr397) phosphorylation was measured with western blot. **(D–G)** Migration and **(H–K)** tube formation of HUAECs transfected with SgRNA or Unc5b gRNA were determined with scratch, transwell, and Matrigel tube-formation assays. The relative number of tubes, tube area, and tube length was analyzed with Image J. Scale bar, 100 µm. Data shown are representative of experimental and quantitative results. *N* ≥ 4 independent experiments. Bars represent mean ± SD. *P < 0.05, **P < 0.01, ***P < 0.001.

To verify the role of Unc5b in the inhibition effect of RGMa on p-FAK signaling, the knockout cells for Unc5b were established with two kinds of plasmids containing different RNAG sequences (RNAG1 and RNAG 2). Both of these effectively knocked out Unc5b, whereas scrambled RNAG (negative control) was ineffective at reducing Unc5b expression (Figure 4A). Using this knockout technique, we observed that Unc5b knockout significantly blocked the decreased *p-FAK (Tyr397)* levels induced by RGMa (*P* < 0.01), as shown in Figures 4B,C. EC migration distance (*P* < 0.05) (Figures 4D,E), EC migration number (Figures 4F,G), and tube formation (*P* < 0.05) (Figures 4H–K) were significantly decreased by RGMa in HUAECs transfected with SgRNA, but this inhibition effect was completely blocked by Unc5b knockout. Unc5b knockout increased EC migration distance, EC migration number, and tube-formation ability compared to ECs transfected with SgRNA. These results indicate that Unc5b is involved in RGMa-induced p-FAK (pY397) decrease and EC inactivation.

### RGMa Affected F-Actin Reassembly and Attenuated the Formation of Filopodia and Lamellipodia *via* Neogenin and Unc5b

**Figure 5:**
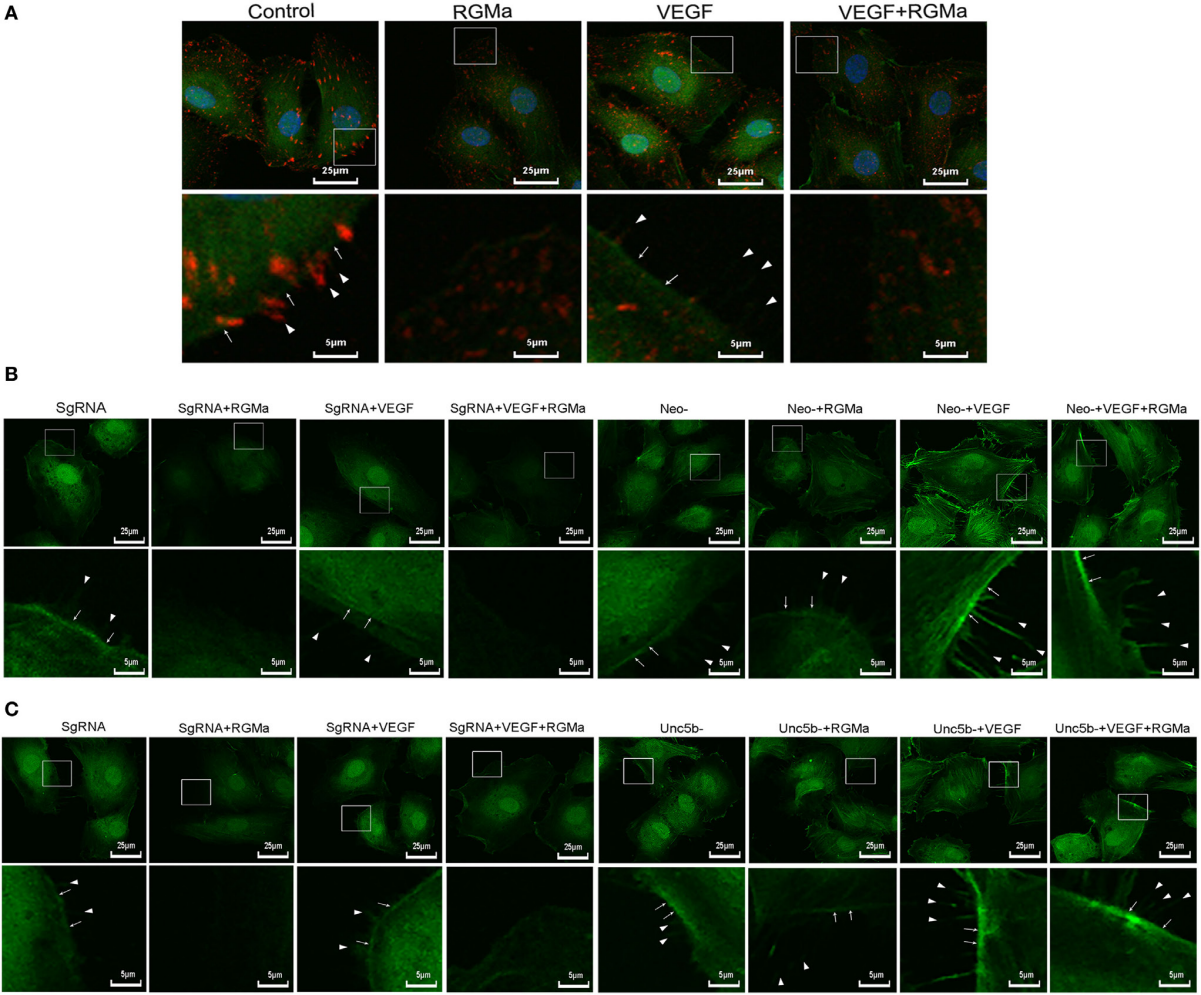
Repulsive guidance molecule a (RGMa) inhibited cytoskeleton reassembly, filopodia, and lamellipodia formation in human umbilical artery endothelial cells (HUAECs) *via* neogenin and Unc5b. Before the immunofluorescence experiment, HUAECs were treated with vehicle, RGMa, VEGF, or VEGF plus RGMa for 40 min. F-actin was stained with phalloidin conjugated with FITC and phosphorylated focal adhesion kinase (p-FAK) connected with primary antibody was labeled with Alexa Fluor 555 donkey anti-rabbit (H + L) secondary antibody. **(A)** Immunofluorescence showed the cytoskeleton (green) change and p-FAK (red) distribution. **(B)** Immunofluorescence showing the cytoskeleton change of HUAECs transfected with SgRNA or neogenin gRNA. **(C)** Immunofluorescence showed the cytoskeleton change of HUAECs transfected with SgRNA or Unc5b gRNA. The filopodia are indicated as sharp spikes (arrowhead), and lamellipodia (arrow) are indicated as flat intensive staining. The merged images are shown in the upper panels, and the amplified indicated areas are shown in the lower panel for different groups. Photographs were obtained with laser scanning confocal microscopy (Nikon, A1 + R, magnification 400×). Results shown are representative images of ≥4 independent experiments.

To further explore the effects of RGMa on angiogenesis and cytoskeleton reassembly, phosphorylated FAK proteins were fluorescently labeled in ECs. Both the intensity and the distribution of phosphorylated FAK on filopodia were downregulated by the addition of RGMa in the control and VEGF groups, with simultaneously attenuated formation of filopodia and lamellipodia (Figure 5A). The inhibition effects of RGMa on the formation of filopodia and lamellipodia were blocked by neogenin or Unc5b knockout (Figures 5B,C).

## Discussion

Angiogenesis is an ideal therapeutic strategy for cerebral ischemic disease. Manipulating the microenvironment can affect angiogenesis in adjacent ischemic areas. RGMa levels were significantly increased and related to neuronal functional recovery in our previous study using a MCAO/reperfusion rat model. We predicted that RGMa may be a regulator of angiogenesis, with effects similar to those of other neuron guidance molecules, and may affect the prognosis of cerebral ischemic injury. In this study, we found that the addition of RGMa significantly downregulated VEGFA levels in ECs and culture supernatants. In contrast, the application of VEGF upregulated RGMa mRNA and protein levels in ECs. RGMa significantly suppressed the proliferation, migration, tube formation, and downregulated the level of p-FAK (Tyr397) in ECs independent of VEGF. Furthermore, RGMa attenuated the formation of filopodia and lamellipodia. All of these effects of RGMa on HUAECs depended on the participation of neogenin receptors and Unc5b co-receptors.

In this study, RGMa mRNA (Figures 1G–I) and protein levels (Figures 1J–L) were significantly upregulated in VEGF-stimulated ECs compared to non-stimulated ECs. A previous study suggested that VEGF might increase the expression of antiangiogenic factors, such as thrombospondin, NPC-2, or netrin-4 (21). ECs expressed more endogenous negative regulators of angiogenesis when exposed to positive regulators of angiogenesis, such as TSP-1 and vasohibin (24, 25). These results indicated that RGMa may be a negative feedback regulator in angiogenesis.

Herein, we observed that RGMa significantly reduced VEGFA levels in ECs (Figures 2A,B) and culture supernatants (Figures 2C–E). Proliferation, migration, tube formation, and p-FAK (Tyr397) were significantly decreased by the addition of RGMa to ECs with or without VEGF stimulation (Figures 2F–X). VEGF can stimulate angiogenesis and reduce infarct size in rats after focal cerebral ischemia (26). Angiogenesis induced by VEGF in rats was associated with reduced neurological deficits after focal cerebral ischemia (27). Therefore, increased RGMa after acute cerebral ischemia may inhibit angiogenesis and recovery of neural networks by downregulating VEGF expression. FAK is a critical mediator for EC cytoskeletal rearrangement, cell migration, and proliferation, which are vital for angiogenesis (28–30). Netrin-4 also inhibited angiogenesis *via* downregulating the level of p-FAK (Tyr397) (21). These results suggest that RGMa might inhibit angiogenesis mediated by VEGF by downregulating VEGF expression and p-FAK (Tyr397) levels *in vitro*.

Using scratch assays and transwell assays, respectively, we revealed that RGMa inhibited the migration ability of ECs. However, Lah and Key reported that RGMa stimulated cell migration and adhesion through its von Willebrand factor type D (vWF) and RGD domain. They also revealed that manipulating the levels of RGMa *in vivo* caused significant migration defects during xenopus gastrulation (6). O’Leary et al. found that RGMa was a repulsive guidance factor for newborn interneuron migration, which can be abrogated by the netrin-1 gradient (31). These findings suggest that the role of RGMa may be different or even contradictory in different cells.

Using a Matrigel tube-formation assay, we showed that RGMa inhibited tube formation in ECs (Figures 2U–X). This effect was abrogated by depletion of neogenin and Unc5b (Figures 3H–K and 4H–K). RGMa induced the same changing trends in tube area and length, indicating that RGMa reduced the number of tubes without changing their diameter. Netrin-1, a neural guidance molecule, inhibited Tyr397 phosphorylation of FAK and angiogenesis induced by VEGF through neogenin and Unc5b receptors (21). Neogenin and Unc5b are also the receptor and co-receptor, respectively, for RGMa. These results suggested that the effects of RGMa on angiogenesis relied on its receptor neogenin and co-receptor Unc5b, which were also involved in RGMa-induced axonal collapse. Our previous study showed that RhoA activation is involved in the repulsive cue of RGMa (10). Interestingly, Brad et al. reported that the activation of RhoA is essential for several aspects of VEGF-induced angiogenesis (14), including EC survival, migration, and permeability. Our study suggested that the addition of RGMa suppressed migration and tube formation in ECs stimulated with or without VEGF, but a previous study showed that RGMa induced growth-cone collapse through RhoA activation (10, 23). This seems to contradict our present findings. However, Oser et al. found that increased RhoA activity can promote cytoskeleton and tube formation in ECs, but has nothing to do with cell proliferation and migration (32). Based on the evidence, we propose that the inhibition effect of RGMa on tube formation may be the result of equilibrium between activated RhoA and downregulated p-FAK. With this in mind, further studies are needed to explore the role of RhoA in RGMa-induced antiangiogenesis.

We showed that the addition of exogenous RGMa significantly inhibited F-actin cytoskeleton reassembly, with decreased filopodia and lamellipodia (Figure 5A). Actin cytoskeleton reorganization is essential for cell migration and chemotaxis (33). Filopodia are actin-rich cell membrane protrusions (34), while lamellipodia are broad, flat cell protrusions consisting of branched actin filaments, which develop at the leading edge of migrating cells. The formation of stress fibers and contractibility of ECs are vital for cell migration and angiogenesis (35, 36). These changes induced by treatment with RGMa were reversed by the knockout of neogenin or Unc5b, indicating that RGMa regulates the formation of stress fibers *via* its receptor neogenin (Figure 5B) and co-receptor Unc5b (Figure 5C).

We also found that the addition of RGMa significantly attenuated FAK phosphorylation at the protrusions of HUAECs. Previous research demonstrated that FAK was required for angiogenesis *in vivo* and VEGF-induced angiogenesis *in vitro* (28). Many prior studies showed that different effects of FAK were partly associated with its localization and specific sites tyrosine phosphorylation (37–39). These results indicate that RGMa inhibits angiogenesis *via* downregulating p-FAK (Tyr397).

In this study, the effect of RGMa on angiogenesis was observed in ECs with or without VEGF stimulation. The results suggested that RGMa may be an antagonist of both endogenous and exogenous VEGF. We will perform further studies to evaluate the effect of endogenous RGMa on angiogenesis.

Taken together, our data demonstrated that RGMa induced decrease of p-FAK (Tyr397) and the number of F-actin stress fibers, filopodia, and lamellipodia. These events suppressed EC motility and tube formation. Numerous pieces of evidence demonstrate the important role of angiogenesis in neural-network recovery after ischemic stroke (40–43). We anticipate that better understanding of the mechanisms of RGMa in angiogenesis may help the discovery of novel and efficient strategies aimed at promoting neural function recovery after cerebral ischemia.

## Materials and Methods

### EC Lines and Reagents

Human umbilical artery endothelial cells were purchased from Sciencell (Sciencell Research Laboratory, USA) and cultured in endothelial culture medium (ECM) supplemented with 5% FBS and 1% penicillin and streptomycin. HUVECs were purchased from Chi Scientific (Jiangsu, China), and RBMECs were purchased from Beijing Dingguo Changsheng Biotechnology (Beijing, China). Recombinant human RGMa, recombinant rat RGMa, recombinant human VEGF, and recombinant rat VEGF were obtained from R&D Systems (MN, USA). Anti-neogenin polyclonal antibody was purchased from Santa Cruz Biotechnology (CA, USA). Anti-FAK monoclonal antibody, anti-FAK (pY397) antibody, anti-RGMa antibody, anti-VEGF, and anti-Unc5b antibody were purchased from Abcam (CB, UK). Anti-GAPDH antibody was obtained from GoodHere (Hangzhou, China). FITC-conjugated phalloidin was purchased from Sigma (Darmstadt, Germany). VEGFA ELISA assay kits were obtained from Cloud Clone Corp. (TX, USA). CRISPR/Cas9 knockout kits for neogenin and Unc5b and TurboFectin 8.0 were purchased from Origene (MD, USA). HUAECs were transfected with neogenin and Unc5b gRNA and then subcultured for five to six passages and purified with puromycin (10 µg/ml), according to the manufacturer’s instructions. The cells were then identified with western blot. The target sequences of neogenin and Unc5b were as follows:

**Table:** 

Target gene		gRNA sequence
Neogenin	KN216942G1	AGGGGGTGCTGAGGAGTCGC
Neogenin	KN216942G2	CTGCTCGGGCGCCGGGCGCC
Unc5b	KN218267G1	AGGTAGGAAGCGATCGGGTC
Unc5b	KN218267G2	GCGCGCCCCGAGCTCCGCTC

### Quantitative Real-time Polymerase Chain Reaction (QRT-PCR)

Reactions were performed using SYBR green PCR Master Mix (Takara Biotech, Dalian, China) in a real-time PCR apparatus (iCycler iQ5, Bio-Rad, Hercules, CA, USA). Sense primer 5′-GACAAACTGACCTGTATGAGAGG-3′ and antisense primer 5′-ACATCTACGCGTCTAGCAGAACAC-3′ were used to amplify the hRGMa-F gene; sense primer 5′-AACTCTGAGTTCTGGAGCGCCAC-3′ and antisense primer 5′-AAAGTCCTGAGGTGTGGGTCCC-3′ were used to amplify the rRGMa-F gene; glyceraldehyde-3-phosphate dehydrogenase (hGAPDH: sense primer 5′-ACCACAGTCCATGCCATCCAC-3′ and antisense primer 5′-TCCACCACCCTGTTGCTGTA-3′; rGAPDH: sense primer 5′-CCATGTTCGTCATGGGTGTGAACCA-3′ and antisense primer 5′-GCCAGTAGAGGCAGGGATGATGTTC-3′) were used as internal controls. Primers were synthesized by Sangon Biotech Co., Ltd. (Shanghai, China). QRT-PCR was performed in strict accordance with the kit instructions. Thermal cycling for all reactions was initiated with a denaturation step at 95°C for 10 min, followed by 45 cycles at 95°C for 15 s and 60°C for 60 s. Each sample from one set of cells was performed in quadruplicate. Three sets of cells were used for QRT-PCR assays. Results were quantitated using the comparative cycle threshold (CT) method 2^−ΔCT^ (44). All calculated concentrations of the target gene were divided by the endogenous reference (GAPDH) to obtain normalized RGMa expression values.

### VEGFA ELISA Kit Assays

Endothelial cells (HUAECs/HUVECs/RBMECs) were treated with or without RGMa. The culture medium was collected at 30, 60, and 120 min, the precipitate was removed by centrifugation, and the clear supernatant extract was analyzed for VEGFA. A sandwich ELISA kit was used to quantify supernatant VEGFA levels. All reagents, samples, and standards were prepared ahead of schedule. Then, 100 µl of standard or sample were added to each well, coated with specific antibodies to VEGFA, incubated for 1 h at 37°C. Next, 100 µl of prepared Detection Reagent A was added, then incubated for 1 h at 37°C. Reagent A was aspirated, and each well was washed three times, then 100 µl of prepared Detection Reagent B was added to each well and incubated for 1 h at 37°C. Reagent B was then aspirated, and each well was washed five times. Next, 90 µl of the substrate solution was added to each well, and the samples were incubated for 10–20 min at 37°C. Finally, 50 µl of the stop solution was added to each well. The assay plate was read immediately at 450 nm. Samples were analyzed in triplicate, and average optical density (OD) values were used to determine concentrations. The ELISA kit assays were performed on three sets of cells. The VEGFA ELISA kit has previously been validated.

### Cell Proliferation Assay

#### Cell-Counting Kit-8 (CCK-8)

Endothelial cells were seeded on 96-well plates (2 × 10^3^ cells/well) and cultured in the complete endothelial culture medium (ECM) for 24 h. The ECs were then treated with vehicle, RGMa (2 µg/ml), VEGF (50 ng/ml), or VEGF plus RGMa, and cultured at 37°C in 5% CO_2_ for another 24 h in the incubator. Then, 10 µl CCK-8 solution (Beyotime, Shanghai, China) was added to each well. The cells were incubated for another 1 h. The absorbance was measured immediately (OD at 420 nm). The relative growth rate was calculated as treatment absorbance/untreated control absorbance. Every group had four wells using the same set of cells. Three sets of cells were used for CCK-8 assays.

#### 5-Ethynyl-2′-Deoxyuridine (EdU) Assay Kit

Human umbilical artery endothelial cells were seeded on 96-well plates (2 × 10^3^ cells/well) and cultured in the complete ECM for 24 h. The ECs were then treated with vehicle, RGMa (2 µg/ml), VEGF (50 ng/ml), or VEGF plus RGMa, and to each well was added 10 μl of EdU (10 mM/ml) at the same time. After 2 h of incubation, the cells were washed with phosphate-buffered saline (PBS), fixed with 3.7% formaldehyde, and permeabilized with 0.5% Triton X-100 at room temperature. The EdU and nucleus were stained with KeyFlour 594 azide and Hoechst 23342, respectively, according to the manual. Images were taken with a fluorescence microscope (Tokyo, Japan) and analyzed with Image-Pro Plus 6.0. The EdU assays were performed in triplicates using three sets of cells. Each group contains three wells, and four areas in each well were taken randomized for statistical analysis.

#### Scratch Assay

Endothelial cells were seeded into 6-well plates to grow for 24 h and 100% confluence. They were then cultured in DMEM supplemented with 0.2% FBS for another 24 h. The cells were wounded with 10 μl sterile pipette tips and cultured in serum-free DMEM with vehicle, RGMa, VEGF, or VEGF plus RGMa. Images were captured (Nikon, Tokyo, Japan; magnification 40×) after incubation for 12 h at 37°C in a 95%:5% (v/v) mixture of air and CO_2_. Ten images were taken randomized from each group for analysis. The scratch assays were performed in triplicate using three sets of cells.

#### Transwell Assay

Transwell cell-culture chamber inserts (polycarbonate, tissue culture-treated, 6.5-mm diameter, 8-µm pore size; Corning Costar, Corning, NY, USA) were used for migration assays. ECs were starved in DMEM medium containing 0.2% FBS for 24 h. Next, 2 × 10^5^ cells/ml were transferred into the upper inserts and cultured in DMEM supplemented with 0.2% FBS at 37°C for 24 h. DMEM supplemented with 0.2% FBS was added to the lower chamber, then the cells were supplemented with vehicle, RGMa (2 µg/ml), VEGF (50 ng/ml), or VEGF plus RGMa. After 18 h, cells were fixed with 3.7% formaldehyde in PBS, and the transwell filters were incubated for 20 min in methanol for permeability, then stained with 1% crystal violet in PBS for 20 min. After being washed three times with PBS, cells on the upper surface filter (non-migrating cells) were removed. Images of cells on the lower surface were acquired with a microscope (Nikon, Tokyo, Japan; magnification 100×), and the numbers of cells were counted using Image-Pro Plus 6.0. Photographs of 10 representative fields were taken randomized from each group for analysis. The transwell assays were performed in triplicate using three sets of cells.

#### Tube-Formation Assay

Matrigel (Corning, NY, USA) was thawed on ice overnight and diluted in DMEM at a ratio of 2:3. Each well of the 96-well plates was coated with 50 µl of diluted Matrigel and incubated at 37°C in 5% CO_2_ for 1 h. HUAECs were cultured in ECM supplemented with 0.2% FBS for 24 h before being used for tube formation. Cells were counted and resuspended to a final concentration of 7.5 × 10^6^ cells/ml in DMEM supplemented with 10% FBS. The resuspended ECs were treated with vehicle, RGMa (2 µg/ml), VEGF (50 ng/ml), or VEGF plus RGMa. The cells were then seeded in 96-well plates coated with Matrigel and further incubated for 3–4 h in an incubator. Images were acquired with a microscope (magnification 40×) connected to a digital camera (D7200, Nikon, Tokyo, Japan). Photographs of 10 representative fields were used for quantification analysis using Image J. Three images were taken randomized from each group for analysis. The tube-formation assays were performed in triplicate using three sets of cells.

#### Western Blotting

To study the expression of VEGF, RGMa, and phosphorylated FAK, ECs were stimulated with vehicle, RGMa (2 µg/ml), VEGF (50 ng/ml), or VEGF plus RGMa for 40 min. Cells were lysed using a mixture containing RIPA (Beyotime, Shanghai, China), PMSF, a protease inhibitor, and a phosphatase inhibitor cocktail (Solarbio, Beijing, China). The homogenate was centrifuged at 10,000 *g* for 10 min. Protein concentration was measured using a bicinchoninic acid protein assay kit (Beyotime, Shanghai, China). Cell lysates were boiled in sample buffer for 5 min, and proteins were separated by SDS-polyacrylamide gel electrophoresis and transferred onto polyvinylidene difluoride membranes (Millipore, Billerica, MA, USA). The membranes were blocked with 5% non-fat dry milk (BIO-RAD, USA) in tris-buffered saline containing Tween 20 (TBST) and incubated for 1 h at room temperature. The primary antibodies, including anti-FAK antibody (1:1,000), anti-FAK (pY397) antibody (1:1,000), anti-VEGFA antibody (1:200), anti-RGMa antibody (1:1,000, Abcam), and anti-GAPDH antibody (1:2,000) were diluted in TBST, the membranes were incubated in primary antibody solutions overnight at 4°C. After being washed in TBST, the membrane was incubated with horseradish peroxidase-conjugated anti-rabbit IgG antibody (Abcam, UK) for 1 h at room temperature. Signals were detected with an enhanced chemiluminescence system (FusionFX, France) and quantified using Fusion Software, according to the manufacturer’s specifications. The relative phosphorylation levels of FAK were normalized to the signal intensity of total FAK.

#### Immunofluorescence Staining of p-FAK, F-Actin, and Nuclear DNA Staining

Human umbilical artery endothelial cells were layered on polylysine-coated glass-bottomed microwell plates (MatTek, MA, USA) and cultured for 24 h. After treatment with vehicle, RGMa, VEGF, or VEGF plus RGMa, cells were fixed for 15 min at room temperature in PBS with 3.7% formaldehyde. The cells were washed in PBS twice, blocked in 10% goat serum for 1 h, then incubated with anti-FAK (Tyr397) antibody (1:200) at 4°C overnight. After thorough washing with PBS, the cells were incubated with Alexa Fluor 555-labeled donkey anti-rabbit IgG (H + L) for 1 h at room temperature. The cells were then stained with phalloidin conjugated with FITC (10 µmol/l) for 1 h at room temperature, and further incubated with diamidino-2-phenylindole (DAPI, 1:1,000, Beyotime, Shanghai, China) for 15 min and again washed with PBS. FITC-conjugated phalloidin, Alexa Fluor 555-labeled primary anti-FAK (Tyr397) antibody, and DAPI were used to stain F-actin and nuclear DNA, respectively, to reveal the location of the cytoplasm and microvilli in the HUAECs. The staining results were imaged using a Nikon AL90 laser confocal scanning microscope (Tokyo, Japan; magnification 400×).

#### Statistical Analysis

The data were normalized to control values and reported as percentages of baseline values (mean ± SD) for ≥3 independent experiments. Student’s *t*-test was used for comparing two groups. Multiple comparisons were performed with one-way ANOVA followed by *post hoc* comparison. Tukey’s test was used for the *post hoc* test. The level of significance was set at *P* < 0.05.

## Author Contributions

Category 1—conception and design of study: XQ, GZ, and YW; acquisition of data: GZ and RW; analysis and/or interpretation of data: GZ, RW, XQ, and RZ. Category 2—drafting the manuscript: GZ, RW, KC, and QL; revising the manuscript critically for important intellectual content: XQ, GZ, RW, and KC. Category 3—approval of the version of the manuscript to be published (the names of all authors must be listed): GZ, RW, KC, QL, YW, RZ, and XQ.

## Conflict of Interest Statement

The authors declare that the research was conducted in the absence of any commercial or financial relationships that could be construed as a potential conflict of interest.

## Abbreviations

RGMa, repulsive guidance molecule a; HUAEC, primary human umbilical artery endothelial cell; HUVEC, human umbilical vein endothelial cell; RBMEC, rat brain microvascular endothelial cell; VEGF, vascular endothelial growth factor; ECM, endothelial culture medium; EdU, 5-ethynyl-2′-deoxyuridine; PBS, phosphate-buffered saline; BCA, bicinchoninic acid; FAK, focal adhesion kinase; DAPI, diamidino-2-phenylindole; CCK, cell-counting kit; NDM, non-fat dry milk; TBST, tris-buffered saline containing Tween 20.
